# Hospital surface disinfection using ultraviolet germicidal irradiation technology: A review

**DOI:** 10.1049/htl2.12032

**Published:** 2022-05-28

**Authors:** Robert Scott, Lovleen Tina Joshi, Conor McGinn

**Affiliations:** ^1^ Department of Mechanical, Manufacturing, and Biomedical Engineering Trinity College Dublin Dublin Ireland; ^2^ Akara Robotics Dublin Ireland; ^3^ School of Biomedical Sciences University of Plymouth UK

## Abstract

Ultraviolet germicidal irradiation (UVGI) technologies have emerged as a promising alternative to biocides as a means of surface disinfection in hospitals and other healthcare settings. This paper reviews the methods used by researchers and clinicians in deploying and evaluating the efficacy of UVGI technology. The type of UVGI technology used, the clinical setting where the device was deployed, and the methods of environmental testing that the researchers followed are investigated. The findings suggest that clinical UVGI deployments have been growing steadily since 2010 and have increased dramatically since the start of the COVID‐19 pandemic. Hardware platforms and operating procedures vary considerably between studies. Most studies measure efficacy of the technology based on the objective measurement of bacterial bioburden reduction; however, studies conducted over longer durations have examined the impact of UVGI on the reduction of healthcare associated infections (HCAIs). Future trends include increased automation and the use of UVGI technologies that are safer for use around people. Although existing evidence seems to support the efficacy of UVGI as a tool capable of reducing HCAIs, more research is needed to measure the magnitude of these effects and to establish recommended best practices.

## 1 INTRODUCTION

Ultraviolet germicidal irradiation (UVGI) is defined as the use of ultraviolet (UV) light in the germicidal range (wavelengths: 200–320 nm) for the disinfection of air and surfaces; UVGI is distinct from the non‐germicidal UVA wavelengths of black lights and suntan lamps (320–400 nm) [1]. The first scientific reports describing the germicidal properties of ultraviolet radiation can be traced back to the nineteenth century, when Downes and Blunt [2] observed that bacteria could be inactivated by direct sunlight. The first installation of UVGI in a hospital was recorded in 1936, when an overhead UV system was installed to disinfect air in operating room settings [3]. The US Center for Disease Control (CDC) first formally endorsed UVGI use in hospitals in 2003 as a supplemental means of water and air sanitization [4]. In 2019, the CDC Guideline for Disinfection and Sterilization in Healthcare Facilities expanded the scope of UVGI to include surfaces: “*the application of UV radiation in the health‐care environment (i.e., operating rooms, isolation rooms, and biologic safety cabinets) is limited to destruction of airborne organisms or inactivation of microorganisms on surfaces*.”[5].

**FIGURE 1:**
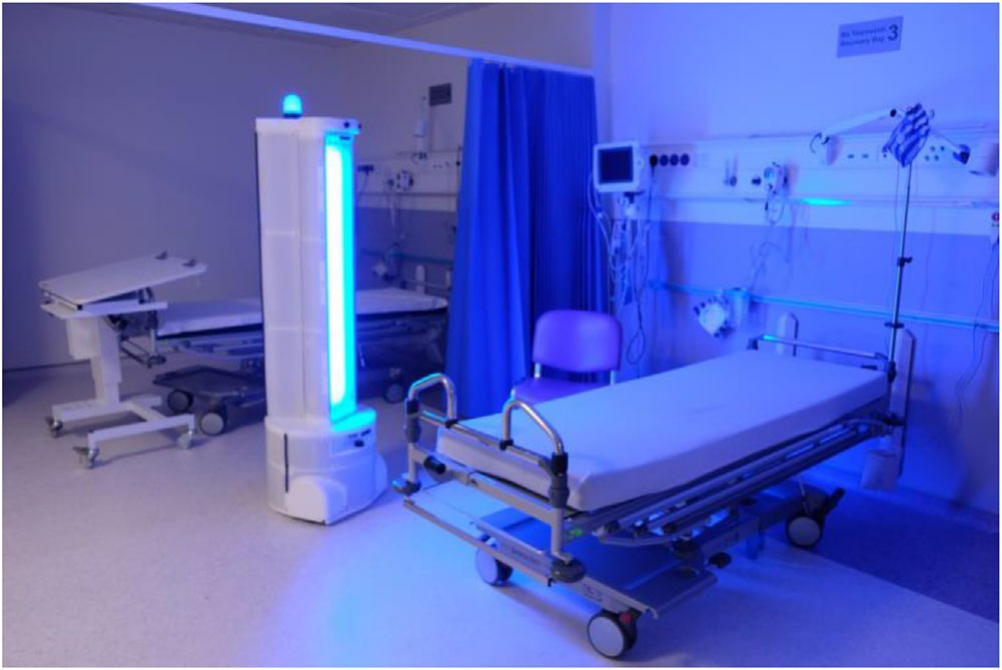
Example of a robotic UVGI platform being used to disinfect surfaces in a hospital

There are currently no harmonized European or international standards for measuring the efficacy of room decontamination using UVGI technologies. The two most applicable standards are the French norm NF T72‐281:2014 “Methods of airborne disinfection of surfaces” and the US norm ASTM E3135‐18 “Standard Practice for Determining Antimicrobial Efficacy of Ultraviolet Germicidal Irradiation Against Microorganisms on Carriers with Simulated Soil”. Unfortunately, neither of these standards are suitable for evaluating the microbiological efficacy of mobile UV devices [6], such as that shown in Figure 1. The occupational safety requirements of UVGI is outlined in EU Directive 2006/25/EC, which provides formulae to calculate the maximum effective radiant exposure that a person can be subjected to over an 8 hour period. In the US, UVGI devices are regulated by the Environmental Protection Agency (EPA) as pesticide devices under the Federal Insecticide, Fungicide, and Rodenticide Act (FIFRA). However, unlike with chemical disinfectants, the EPA does not routinely review the safety or efficacy of UV light devices^1^.

In the absence of formal guidance on the recommended procedures for deploying UVGI technology, there is a need to establish best practice from the currently available published literature. In Section 2, we describe the methodology we followed to conduct the review. Next, we segment the field based on the type of UVGI technology, the clinical setting where it was deployed, and the experimental design that was followed, and provide observations on the best practices in each. Finally, we conclude by identifying the key limitations of the study and suggest directions for future research.

## 2 MATERIALS AND METHODS

The literature search for the review was carried out in March 2021. The first step involved identifying clinical studies that used UVGI technology. Literature searchers were conducted using SCOPUS, PubMed and Google Scholar. The search involved using multiple keywords using the terms ‘UV’, ‘ultraviolet’, ‘UVGI’ with qualifiers including ‘disinfection’, ‘clinical’, and ‘hospital’ in various combinations.

**FIGURE 2:**
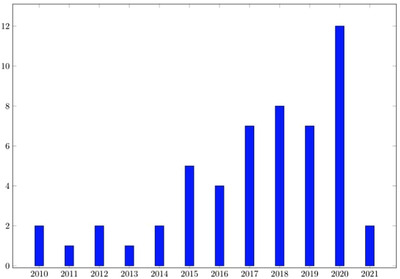
Distribution of clinical UVGI studies considered in our review by year (date of search 14 March 2021)

The review consisted of a 3‐stage process. In the first stage, we undertook a broad keyword search using the keywords outlined above, which returned 134 papers. The second stage involved reading all abstracts and removing wholly non‐relevant papers such as review papers [7, 8, 9], those that involved in vitro experiments or decontamination chambers [10, 11, 12], papers not published in English, such as [13], or those unavailable for download [14]. The third stage involved re‐reading the abstracts and narrowing down the papers to those that appeared to consist of real‐world studies involving room decontamination using UVGI devices. Papers removed at this stage included studies using light outside the UV spectrum [15], where the UVGI device was used to disinfect water systems [16, 17], where the device comprised a closed chamber targeted primarily at specific components (such as dental moulds [18, 19] or stethoscopes [20]), rather than full room disinfection. This led to a final selection of 53 papers being chosen for study in the analysis. A frequency analysis showing the distribution of the shortlisted papers by year is given in Figure 2. It is apparent from this chart that clinical application of UVGI technology has been growing since 2010, but has increased considerably since the onset of the COVID‐19 pandemic, with the number of publications in 2020 far exceeding that of each of the previous three years.

## 3 RESULTS

Papers were examined under the following headings: UVGI technology, clinical settings where UVGI was used, and experimental design.

### 3.1 UVGI technology

For each paper reviewed, we identified the core UV‐generating technology that was used (Table 1). The majority of studies (24 papers) utilized devices that produced UV irradiation using pulsed xenon (PX‐UV) technology that emits broadband radiation in the 200 nm‐320 nm spectrum. Devices using low‐pressure mercury lamps (LPML), which produce narrow‐band germicidal irradiation in the UVC spectrum at a wavelength of 254 nm, were also common (20 papers). Only 2 papers investigated the efficacy of so‐called Far‐UV technology, which produces irradiation at 222 nm using KrCl excimer lamps. UV LED's are not yet a widely used technology for full room decontamination and were not found in the shortlisted papers in this review.

**TABLE 1:** Summary of the type of UVGI technology used across the studies in the review

Low pressure mercury lamp (LPML)	Pulsed xenon UV (PX‐UV)	Far‐UV
[28, 29, 30, 31, 32, 33, 34, 35, 36, 37, 38, 39, 40, 41, 42, 43, 44, 45, 46, 47]	[48, 49, 50, 51, 52, 53, 54, 55, 56, 57, 58, 59, 60, 61, 62, 63, 64, 65, 66, 36, 67, 68, 69, 70]	[71, 72]

Several papers described the use of UVGI in clinical settings without describing the underlying technology that was used [21, 22, 23, 24, 25, 26] and were not included in Tables 1 or 2. While [27] utilised a UV robot, the type of technology is unclear and was also omitted from Table 1.

**FIGURE 3:**
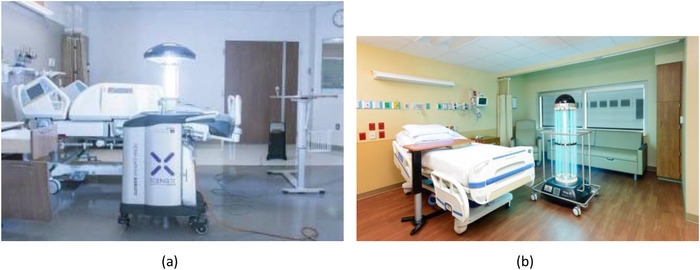
Examples of UVGI devices that appear frequently in literature: (a) the Xenex LightStrike mobile device, a PX‐UV device, and (b) the Tru‐D mobile device, a LPML device

Next, we examined how the devices were deployed operationally during UVGI treatment. Of the devices that appeared in our review, nearly all of them were manually operated and needed to be pushed in place (41 papers) and the most widely used device was the Xenex Lightstrike (Figure 3a) PX‐UV system (18 papers). The second most widely used devices were the Tru‐D (Figure 3b) (5 papers) and Skytron LPML systems (3 papers). Four papers used devices that were manually waved over the surface to be disinfected. The remaining devices were static, such as upper room fixtures (2 papers), or fully autonomous robotic devices that were able to automatically navigate to different waypoints during UVGI with minimal human intervention (1 paper). A breakdown of the degree of mobility of the devices used in each paper is given in Table 2.

**TABLE 2:** The degree of mobility of the UVGI devices used in each study

Fixed/Static	Manual (push‐in‐place)	Manual (handheld)	Autonomous
[42, 44]	[48, 49, 28, 50, 51, 52, 53, 54, 55, 56, 30, 31, 57, 32, 33, 58, 34, 59, 60, 61, 35, 62, 63, 64, 65, 66, 37, 67, 68, 38, 39, 69, 70, 40, 27, 41, 43, 44, 45, 46, 47]	[71, 72, 36, 46]	[29]

### 3.2 Clinical settings where UVGI is used

To determine where UV disinfection has been most applicable, we examined the range of clinical settings where UVGI deployments have taken place (Table 3). It was observed that UVGI treatments were nearly always performed in rooms that did not have patients present to mitigate the risk that staff/patients might be exposed to hazardous levels of UV irradiation during the UVGI procedure [33, 56, 69]. Patient rooms were found to be the most common location for UVGI (30 papers), both for non‐isolation patients and patients isolating with an infection. These settings typically consisted of a bed, a bathroom, and high touch surfaces such as bed rails, bed control panels, call button, tables, and door handles [63, 70].

**TABLE 3:** The most common clinical settings where UVGI technology was used

Patient rooms	ICU	OR	Other
[28, 52, 21, 53, 54, 55, 56, 71, 30, 31, 57, 32, 33, 22, 35, 62, 63, 64, 66, 37, 67, 68, 39, 69, 70, 40, 25, 43, 44, 47]	[48, 28, 55, 58, 60, 62, 36, 47]	[55, 34, 59, 60, 38, 69, 41, 42]	[49, 28, 29, 50, 51, 58, 72, 59, 60, 61, 65, 36, 23, 69, 24, 27, 45, 26, 46, 47]

Intensive care units (ICUs) and operating rooms (ORs) were the second most common setting for UVGI (8 papers each). ICUs usually consisted of patient beds, bed rails, cardiopulmonary monitors, ventilators, and other medical equipment such as keyboards and cart handles [48, 58]. Operating rooms commonly included a surgical table, anaesthetic machines and support equipment. High touch surfaces in these rooms include tray tables, monitors, infusion pumps, and scialitic lamps [55].

Other settings where the applicability of UVGI has been evaluated include burn units [69], hyperbaric chambers [27], radiology rooms [29], oncology units [35], and clinical labs within a hospital [50]. Use of UVGI technologies in hallways or other public areas was only reported in a small number of studies [28, 46]. Where the setting was not specifically named [24, 26], or where various surfaces are described but the room was not named [46], the study was classified in the "Other" column of Table 3.

### 3.3 Experimental design in UVGI studies

The impact of UVGI as an infection control tool has been examined using two main approaches: (1) direct estimation of bioburden reduction, and (2) the reduction of healthcare acquired infections (HCAIs) following UVGI intervention.

Based on our review, we found that the metric is generally dependent on the study duration, which is summarised in Table 4.

**TABLE 4:** The duration of UVGI studies in the literature

Short term	Medium term	Long term
[49, 50, 51, 56, 71, 30, 72, 60, 63, 66, 36, 38, 39, 24, 40, 27, 42, 73, 43, 26]	[29, 53, 54, 55, 31, 32, 33, 58, 34, 37, 67, 70, 44, 46]	[48, 28, 52, 21, 57, 59, 61, 22, 35, 62, 64, 65, 68, 23, 69, 41, 45, 47]

For the purpose of our analysis, short‐term trials were characterised by the performance of isolated experiments, typically spanning one or several days of testing. For example, Chen et al. [50] conducted swab sampling to compare bioburden on surfaces before and after UVGI in three rooms during a single day of testing. In total, we counted 20 short‐term studies.

Medium‐term studies (14 papers) were defined as periodic environmental sampling over a number of weeks or months during which a UVGI device has been in use. This generally involved more systematic testing that better resembled real‐world deployment conditions, for example, Yang et al. [32] carried out swab testing on a routine basis while the device was integrated into hospital workflow over a 6 month period between October 2015 and March 2016.

Long‐term studies (18 papers) investigated the impact of UVGI devices that had been implemented into the workflow of the clinical setting for more than 12 months and/or employed HCAI reductions as the key metric. For example, Haas et al. [59] reported HCAI reductions following a 22‐month deployment of a UVGI system within a contact precaution unit, operating rooms, dialysis unit and burn victim unit of an acute hospital.

#### 3.3.1 Environmental sampling methods

Measuring bioburden reduction was found to be the primary metric for short and medium‐term trials. To empirically measure or quantify bio‐burden reduction, it is necessary to sample surfaces in the room before and after UVGI. Of the studies reviewed in our analysis, common methods of surface sampling included contact plates, swabs, and sponges. Of the studies reviewed, the majority used a generic medium, such as Tryptone Soya Agar (TSA), which is targeted primarily at bacterial recovery. However, on occasion, selective agars were be used to test for specific bacteria (such as *Clostridioides difficile* [55]) and specific fungi (such as *Aspergillus fumigatus* [32] and *Candida albicans* [46]). None of the studies in the review involved viral recovery, and therefore effectiveness of UVGI at inactivating viruses in clinical settings was not directly measured. A breakdown of the different sampling methods used in the reviewed studies is given in Table 5.

**TABLE 5:** Common methods associated with UVGI testing and validation

Contact plates	Swabs	HCAIs	Other
[48, 49, 29, 52, 54, 55, 31, 33, 58, 34, 72, 60, 66, 38, 69, 70, 27, 43]	[29, 50, 52, 53, 56, 71, 30, 32, 37, 67, 68, 27, 44, 46]	[48, 28, 57, 59, 61, 22, 35, 62, 64, 65, 68, 23, 69, 41, 45, 47]	[49, 55, 63, 36, 68, 23, 39, 69]

##### Contact plate method

The use of contact plates, also known as Replicate Organism Detection And Counting (RODAC) plates, was the most common method of sampling surfaces across UVGI studies (18 papers). Contact plates are suitable for flat surfaces and some curved surfaces (using what is known as a ‘roll plate’ method) [74]. Standard mediums, such as Tryptone Soya Agar (TSA) is typically poured into a plastic contact plate (approximately 5 cm in diameter) and pressed flat against a surface in order for surface microorganisms to stick to the medium. Bioburden is most commonly quantified by counting the number of colony forming units (CFUs) on the plate after a period (typically 24–48 h) of aerobic incubation at 30–37°C [38, 54, 60].

##### Swab method

Another common method of sampling involves using swabs (14 papers). Although they have been shown to be difficult to standardise [75], swabs have the benefit of manipulation around uneven surfaces. Typical sampling procedures involve the use of sterile swabs moistened with sterile saline and rolled on a discretized area on a surface e.g. 5 cm x 5 cm. Analysing swabs is more labour intensive than contact plate methods as it is necessary to transfer microbes from the swabs to a cultivation medium post‐sampling [76].

##### Other sampling methods

Sponges, another indirect sampling method, were observed in a small number of studies [49, 51, 55, 63]. Their use involved a sterile sponge moistened with saline being wiped across a surface and subsequently placed in a bag with Phosphate‐buffered saline (PBS) and processed in a lab blender. The fluid undergoes processing before being poured onto agar plates and incubated overnight before analysis [77]. The recovery of microbes from the sponge can vary and this method is not as commonly applied as swab testing.

One study employed the use of UV sensitivity cards to estimate bioburden reduction [39]. This involves cross‐referencing empirically recorded UV dose readings with standard lookup tables (like those found in [1]) citing the UV inactivation levels for different microbes). While this method can be a useful indicator in optimising the positioning/route of a UV device, it's not sufficient as a standalone metric in determining the disinfection efficacy since the efficacy of UVGI is dependent on a range of factors including the type of microorganism, the material properties of substrate to which the microorganism is attached [78], and a number of other parameters which may vary in real‐world settings. Where possible, therefore, UV sensitivity measurements should be reserved as an adjunct to robust microbial sampling of the environment.

One study, conducted by Rutula et al. [33] evaluated the efficacy of UVGI in an empty patient room using Formica sheets inoculated with a known quantity of vegatative bacteria. Results from these tests were later compared against data from a follow‐on experiment that used contact plate samples taken from a room that housed patients with MRSA infections. Both experiment found statistically significant reductions in CFU count following UVGI.

### 3.4 Correlation between HCAI prevalence and UVGI

The risk of healthcare associated infections (HCAIs) following patient discharge was cited by a number of studies as a major driver to implement novel disinfection technologies. UVGI studies have targeted pathogens responsible for the majority of HCAI fatalities [26], including methicillin‐resistant *Staphylococcus aureus* (MRSA) (21 papers), vancomycin‐resistant *Enterococcus* (VRE) (14 papers) and *Clostridioides difficile* (18 papers). Other pathogens of interest included *Escherichia coli* [36, 43, 44, 46], *Klebsiella pneumoniae* [43, 46, 50, 55] and *Pseudomonas aeruginosa* [36, 43, 50, 69, 44, 46]. The pathogens that were specifically targeted in UVGI studies are summarised in Table 6. This table combines studies that employ direct measurement of HCAI reductions as the key metric as well as studies that carried out environmental sampling and/or UVC level measurement.

**TABLE 6:** The most common pathogens of concern in the literature

MRSA	VRE	Clostridioides difficile	Other
[48, 28, 52, 56, 71, 30, 31, 57, 32, 33, 58, 72, 59, 22, 62, 66, 36, 39, 70, 41, 43]	[49, 28, 53, 56, 71, 30, 31, 32, 33, 59, 61, 22, 62, 65]	[55, 56, 71, 30, 31, 59, 61, 22, 35, 62, 63, 64, 65, 23, 39, 69, 40, 25]	[28, 50, 55, 36, 69, 43, 44, 46]

A total of 16 papers reported the effect of UVGI on HCAI prevalence. Of these studies, 11 papers reported a reduction in HCAIs at a statistically significant level. The majority of papers in this category considered HCAI rates in their totality, rather than types of infection linked to specific microorganisms. With a focus on broad and efficient deployment across patient rooms, Schaffzin et al. [62] reported a 16% reduction in HCAI rates following the introduction of UVGI. Sampathkumar et al. [65] observed a decrease in HCAIs from 28.7 to 11.2 per 10,000 patient days—a 39% decrease in the PX‐UV intervention period—however, the UVGI process added 25 min to the terminal cleaning process. A statistically significant reduction in both HCAI rate and hospilisation rate was noted by Kovach et al. [68] in a 12‐month UV intervention period when compared to the 36‐month period pre‐intervention. Kitagawa et al. [57] also reported statistically significant reductions of MRSA following PX‐UV intervention. A 44% reduction of viral infection rates was reported in a 12‐month study by Pavia et al. [45] while Napolitano et al. [47] observed a 34.2% reduction of HCAIs over an equivalent period. Raggi et al. [28] found a 19.2% of HCAIs of multi‐drug resistant organisms (MDROs) after a 12‐month UVGI intervention period when compared to the pre‐intervention period; emergency department admissions were not adversely affected during this period.

A number of papers explored the correlation between *C. difficile* infections and the introduction of UVGI. Anderson et al. [22] recorded a significant reduction of up to 30% of *C. difficile* infections when terminal disinfection was enhanced with UVGI. Interestingly, no significant reduction was found when the standard protocol was enhanced with use of chlorine releasing compounds, or a combination of chlorine releasing compounds and UV. During a 52‐week intervention period carried out by Pegues et al. [35], the *C. difficile* infection rate declined 25% in UV units while a rise of 16% was observed in non‐UV units. The impact of UVGI on average room cleaning time and room turnaround was negligible. Miller et al. [64] implemented two interventions aimed at reducing *C. difficile* infections. The first intervention involved forming a multidisciplinary team dedicated to reducing HCAIs; this resulted in a reduction of 17% from baseline figures. The introduction of PX‐UV as an adjunct to manual cleaning further reduced the transmission rates by 57%. A 20% reduction of hospital‐acquired MDRO plus *C. difficile* rates was observed by Haas et al. [59] in a 22‐month intervention period when compared to the 30‐month pre‐UV period. The impact of UVGI against MRSA prevalence was examined by Morikane et al. [48] who reported a 29% decrease following the implementation of the technology, as well as a 63% reduction of drug‐resistant *Acinetobacter* acquisition.

Several studies did not measure significant reductions in HCAI following the introduction of UVGI. Brite et al. [61] reported no significant change in VRE and *C. difficile* incidence rates during a 20‐month study period of a transplant unit. The authors note that this is likely caused by the compromised immune systems of transplant patients; they remain highly susceptible to HCAIs despite a reduction in environmental pathogens following the introduction of UVGI. While Green et al. [69] observed reductions in environmental of microorganisms, no significant reductions in HCAI rates were observed. Similarly, Goto et al. [23] observed no statistically significant difference in hospital‐acquired *C. difficile* rates.

## 4 DISCUSSION

Although the efficacy of UVGI disinfection has been established for a long time, examination of the published literature shows that the clinical practice of using UVGI technologies remains fragmented and rapidly evolving. Conventionally, UVGI has found greatest applicability in patient rooms, ICU, and OR settings, however, new applications have emerged in a diversity of settings from radiology to ambulances.

The specifications of UVGI devices vary significantly across the studies examined. In total, more than 17 distinct devices featured. We observed that important experimental parameters (including details on the exact placement of the device) were often not described in sufficient detail for replication and important technical device information was regularily omitted and could not be easily found within references or by examination of product datasheets. Most studies used either PX‐UV technology, which irradiates using pulses across a wide spectrum of UV wavelengths, or LPML, which irradiates continuously across a narrow band of wavelengths in the UVC spectrum (200–280 nm). The use of lower wavelength devices that irradiate at 222 nm wavelengths (so called Far‐UV) is also increasing. The effect of UV wavelength on microbial inactivation is currently unexplored, especially in clinical settings. While studies such as that performed by Cadnum et al. [79] have attempted to investigate performance differences between PX‐UV and LPML technologies, it is not possible to draw strong conclusions from their findings due to the major differences in operating procedures and hardware specifications of the platforms evaluated. While some research has explored the optimal placement of the UVGI devices in the room (most notably the paper by Tiseni et al. [80]), the topic remains under‐explored and would benefit from further investigation. Furthermore, while the majority of UVGI systems required manual positioning, newer platforms that can move autonomously seem to offer potential to reduce the labour requirements of using the technology.

The majority of UVGI studies have involved tests that take place over a single deployment of the technology. This review supports findings from the systematic review conducted by Peters et al. on the effect of environmental hygiene interventions on HCAIs and patient colonization <https://doi.org/10.1186/s13756‐022‐01075‐1> that where UVGI has been implemented over prolonged periods of time, it has often been linked with reductions in HCAI. However, given the many other factors that may confound these findings, there is a need for more systematic bio‐burden measurement during long‐term deployments and for more medium‐to‐long term studies to be conducted. Few studies offer qualitative perspectives of the challenges of integrating UVGI within clinical workflows; this should also be addressed in future work. In the absence of standardized test methods, it is challenging to objectively compare UVGI performance between studies conducted in clinical settings. Factors that affect the ability to make accurate comparisons include: differences in the baseline bio‐burden levels, differences in environmental sampling methods and growth media used in the study, and differences in the timing of when the sampling was carried out. While the development of formal professional standards may not be immediately forthcoming, greater scientific rigor can be achieved if researchers planning future studies prioritize experimental designs that are easily repeatable, involve the use of more than one sampling technique, and use metrics that allows for benchmarking with previous studies in the literature.

Most studies involving environmental sampling used post‐treatment reductions in colony forming units as the primary metric for quantifying efficacy of the UVGI procedure. A limitation of this approach was that the strain of the microbe captured was usually not apparent, especially when the agar media used was generic and promoted the growth of many common bacteria/fungus. Therefore, although the UVGI procedure achieved a reduction in bioburden, the extent to which the treatment was effective against multi‐drug resistant organisms (MDROs) or other specific microbes of interest was unknown. We found one study, carried out by Villacís et al. [60], that overcame this limitation by first measuring CFU reductions after an in‐situ UVGI deployment, and later performed in vitro UVGI testing on four specific strains of MDROs that were identified at the hospital through a process of environmental sampling and PCR testing.

While the disinfection efficacy was the primary focus in most studies, the time taken to perform the procedure was also found to be a measure of high importance [21, 22, 28, 53, 54, 62]. This is due to the demands for patient rooms and specialised units within the hospital, as well as a need to reduce the times taken to disinfect rooms without reducing disinfection efficacy. Raggi et al. [28] observed significant cost‐savings in a US hospital following hospital‐wide UVGI intervention; this was due to a reduction of excessive inpatient stays as a result of HCAIs.

The papers reviewed in this article do not represent an exhaustive list, but we believe it does contain a good representative sample of the current state of the literature in UVGI. The keyword search was biased towards studies that were conducted in clinical environments may have missed studies conducted in other settings where UVGI is reported to be sometimes used (including hospitality, retail etc.). The scope of the study was further limited to surface disinfection, and therefore practices involving the use of UVGI technology for inactivating microbes in air were not investigated.

Finally, despite a body of evidence indicating effectiveness of UVGI against a broad spectrum of pathogens, including microbes that are known to exhibit antimicrobial and biocide resistance, known limitations of UVGI, namely that obstructing objects may cause some surfaces to be shadowed and therefore not to receive the intended UV dose, means that UVGI is most effectively used in conjunction with a biocide‐based disinfection procedure carried out by human cleaning staff. Investigating approaches for optimizing the combined use of biocide and UVGI disinfection regimes appears to be an interesting direction for future research.

## 5 CONCLUSIONS

Ultraviolet germicidal irradiation has been an established means of surface and air disinfection for decades and its applications in clinical settings have been growing steadily in recent years. In this paper, we reviewed the published literature that investigated the deployment of UVGI systems in clinical systems as a means of surface disinfection. We shortlisted 52 papers in total, which were subsequently examined based on the type of UVGI technology used, the clinical settings where they were used and the experimental design that was followed in the study.

PX‐UV and LP‐UV technology was well represented in the literature, however, often important technical information on the devices’ specifications was not publicly available. The integration of autonomous mobility and the use of far‐UV as the UVGI source emerged as high potential technologies, but are currently underrepresented.

The application of UVGI room disinfection systems was limited to settings that could be evacuated during use, since high levels of background UV radiation produced by currently available UVGI devices poses a health and safety risk. Consequently, the technology has found greatest application in individual rooms after patient discharge and in operating room settings as supplementary part of routine disinfection procedures.

The majority of studies were conducted over relatively short time frames and included the empirical measurement of bioburden using standard environmental sampling techniques, whereas long term studies typically utilized HCAI prevalence measured over several months/years as the primary metric. Most of these studies indicated that the introduction of UVGI led to a measurable reduction of HCAIs, however lack of standardisation and the presence of confounding factors necessitates that further studies are required before strong conclusions can be drawn.

## CONFLICT OF INTEREST

Conor McGinn declares a conflict of interest due to his involvement as a shareholder and co‐founder of Akara Robotics, a company involved in the development of UVGI technology.

## ETHICS APPROVAL

N/A.

## PATIENT CONSENT STATEMENT

N/A.

## PERMISSION TO REPRODUCE MATERIAL FROM OTHER SOURCES

Copyright permission is obtained for using the photo in Figure 1. Images used in Figure 3 are reproducible under fair use.

## Data Availability

The data that support the findings of this study are available from the corresponding author upon reasonable request.

## Footnotes

1
https://www.epa.gov/sites/default/files/2020‐10/documents/uvlight‐complianceadvisory.pdf
